# Challenging the opinion that SAPHO syndrome is associated with low intracellular ROS production in neutrophils

**DOI:** 10.1186/1546-0096-13-S1-P16

**Published:** 2015-09-28

**Authors:** P Wekell, H Björnsdottir, L Björkman, M Sundqvist, K Christenson, V Osla, S Berg, A Fasth, A Welin, J Bylund, A Karlsson

**Affiliations:** 1 University of Gothenburg, Dept Pediatrics, Gothenburg, Sweden; 2 University of Gothenburg, Dept Rheumatology and Inflammation Research, Gothenburg, Sweden

## Background

SAPHO syndrome, characterized by synovitis, acne, pustulosis, hyperostosis, and osteitis, belongs to the autoinflammatory bone disorders, in which dysregulation of innate immunity typically causes inflammation in sterile bone. The mechanisms underlying SAPHO syndrome are unknown, but neutrophil activation is suggested as part of disease pathophysiology. Previously, a patient with SAPHO syndrome-like phenotype was shown to lack production of intracellular NADPH-oxidase-derived reactive oxygen species (ROS) in response to phorbol myristate acetate (PMA; Ferguson et al, Arthritis and Rheumatism, 2008). In absence of phagosome-formation, such ROS are produced in intracellular granules, and are suggested to be part of regulatory signaling associated with hyperinflammatory disease.

## Objective

To investigate if aberrant neutrophil intracellular production of NADPH-oxidase-derived reactive oxygen species is a general feature and disease mechanism in SAPHO syndrome.

## Patients and methods

Neutrophil function was explored in a cohort of four patients with SAPHO syndrome, two of which were sampled during both inflammatory and non-inflammatory phase. Intracellular neutrophil reactive oxygen species production was determined by luminol-amplified chemiluminescence in response to PMA.

## Results

Cells from all patients produced normal amounts of reactive oxygen species, both intra- and extracellularly, when compared to internal controls as well as to a large compilation of healthy controls assayed in the laboratory over time (showing an extensive inter-personal variability in a normal population). Further, intracellular production of reactive oxygen species increased during the inflammatory phase. Neutrophil activation markers were comparable between patients and controls.

## Conclusion

Dysfunctional generation of intracellular ROS in neutrophils is not a general feature of SAPHO syndrome. Secondly, serum amyloid A appears to be a more sensitive inflammatory marker than C-reactive protein during improvement and relapses in SAPHO syndrome.

